# Detection of Microplastics in Human Breast Milk and Its Association with Changes in Human Milk Bacterial Microbiota

**DOI:** 10.3390/jcm13144029

**Published:** 2024-07-10

**Authors:** Apisith Saraluck, Tachpon Techarang, Phattarika Bunyapipat, Khununya Boonchuwong, Yupparase Pullaput, Auemphon Mordmuang

**Affiliations:** 1 School of Medicine, Walailak University, Nakhon Si Thammarat 80160, Thailand; apisith.sar@mahidol.ac.th; 2 Department of Obstetrics and Gynaecology, Faculty of Medicine, Ramathibodi Hospital, Mahidol University, Bangkok 10400, Thailand; 3 Department of Tropical Pathology, Faculty of Tropical Medicine, Mahidol University, Bangkok 10400, Thailand; 4 Walailak University Hospital, Walailak University, Nakhon Si Thammarat 80160, Thailand; 5 The Center for Scientific and Technological Equipment, Walailak University, Nakhon Si Thammarat 80160, Thailand

**Keywords:** microplastics, human breast milk, microbiota, women’s health

## Abstract

**Background:** Presently, there is increasing public consciousness regarding the contamination and detection of microplastics (MPs) within the human body, and studies on the detection and characterization of MPs in human breast milk are limited. **Objectives:** This study aims to investigate the prevalence and characteristics of MPs found in human breast milk and examine the relationship between maternal hygiene practices, complications that may arise during breastfeeding, and the composition of the bacterial microbiota. **Methods:** Postpartum breast milk was analyzed for MPs using Raman micro-spectroscopy. The relationship between MP detection, maternal hygiene, breastfeeding complications, and bacterial microbiota was examined. In order to identify correlations and differences between groups that had detected and non-detected MPs, statistical analyses were performed, which involved demographic comparisons and correlation network analysis. **Results:** The mean age of the 59 postpartum women was 28.13 years. We found MPs in 38.98% of breast milk samples (23 of 59), exhibiting diverse morphological and chemical characteristics. Most MP polymers were polypropylene, polyethylene, polystyrene, and polyvinyl chloride. Maternal hygiene and breastfeeding complications differed between the MPs-detected and non-detected groups. Maternal behaviors may influence the presence of microplastics in breast milk, which were associated with these differences. Bacterial microbiota analysis revealed significant taxonomic differences between the MPs-detected and non-detected groups. *Staphylococcus* and *Streptococcus* dominated the MPs-detected group, while *Enterobacter*, *Escherichia*, *Pseudomonas*, and *Acinetobacter* dominated the non-detected group. The MPs-detected group had a more even bacterial distribution, especially Bacteroides. **Conclusions:** This study found MPs in 38.98% of breast milk samples using Raman micro-spectrometry, with PP, PE, and PVC being the most common. Significant differences in maternal hygiene and breastfeeding complications were found between the groups with and without MPs. Breast milk microbiota may be linked to MP detection. Further study should be conducted to identify the possible maternal-child health.

## 1. Introduction

Microplastics (MPs) are plastic byproducts that emerge from the decomposition of plastic constituents present in routine consumer products, including scrubs, cleaning liquids, and cosmetics [1]. The dimensions of MPs may span from nanometers to millimeters. Although particles ranging from 10 to 15 μm are typical, MPs can also be considerably larger or smaller. MPs are exposed to contamination by a wide range of environmental pollutants and compounds. Possible ingestion of these contaminants by organisms, including humans, could result in their release and subsequent entry into the human body. They have the potential to accumulate in tissues and organs once they enter the body [2]. The ability to traverse cellular membranes, enter the human body, and accumulate or be eliminated in accordance with the specific mechanisms of cellular immune responses. Studies have shown that exposure to MPs can lead to inflammation and immune reactions in various organisms, including human, bacteria, and microbiota [3,4]. Currently, medical researchers worldwide are focused on the contamination of MPs within the human body, which may adversely affect human health, weaken the immune system, and disrupt microorganisms. They also aim to increase public awareness of these potential risks. It is well-established that the surrounding environment during infancy may raise an individual’s vulnerability to specific diseases or changes in microorganisms, such as bacterial microbiota, during development [5]. Early development exposure to small concentrations of MPs may disrupt the exchange of gases and nutrients and microorganisms and result in long-term health effects [6]. The initial identification of MPs in the human placenta indicates the potential for a transfer from the mother to the fetus during utero, according to the research that triggered the debate concerning the relationship between MPs, nutrition, and maternal breast milk [7]. Furthermore, MPs have been identified in infant stool and meconium; however, the origins of these plastics remain elusive. Exposures in early life can manifest through various means, including the placenta, lactation (from maternal breast milk), infant formula, feeding bottles, and plastic objects [8]. A recent investigation revealed that, among the 34 samples examined, 26 displayed atypical and spherical fragments that resembled MPs originating from various polymer matrices. The primary MPs identified were polyethylene (PE), polyvinyl chloride (PVC), and polypropylene (PP). Additionally, nitrocellulose, poly-ethyl methacrylate (PEMA), high-density polyethylene (HDPE), polystyrene (PS), polyamide (PA), and acrylonitrile butadiene styrene were found in low concentrations. Nevertheless, this quantity might be grossly underestimated [9]. Furthermore, an additional study documented the identification of sixteen different types of MPs, with PA and polyurethane (PU) emerging as the most prevalent. That study provides interesting insights on MP exposure, including the possibility of MP exposure through water intake, toothpaste or scrub cleanser use, breastfeeding, and the use of feeding bottles and plastic equipment [10]. Despite numerous previous studies reporting the detection of MPs in human tissue, such as the gastrointestinal (GI) tract or skin, and observing the effects of MPs from human tissue or the GI tract on microorganisms, bacteria, and microbiota, research on the detection of MPs in human breast milk remains limited, with only a few of publications to date. In addition, research on breastfeeding complications, maternal behavior, and the risk of MP exposure during antepartum, postpartum, and breastfeeding has very limited data, and there is no study examining the relationship between MP detection and microbiota concentration in human breast milk; this underscores the lack of knowledge in this area of study. Exploring the identification of MPs in human breast milk as well as the effect of MP detection on changes in the bacterial microbiota and maternal behavioral factors associated with MP detection in human breast milk is the purpose of this research initiative.

## 2. Materials and Methods

### 2.1. Study Design and Population

A prospective, multi-center cohort design was used for this pilot observational descriptive research study. The Ethical Committee of Walailak University, Thailand (WUEC-23-046-01) approved this study, which followed the Declaration of Helsinki, a component of the Code of Ethics of the World Medical Association for human subject experiments. From Walailak University Hospital’s Obstetrics and Gynecology Department and Thasala Hospital’s Breastfeeding Clinic in Tha Sala District, Nakhon Si Thammarat Province, Thailand, all patients had uncomplicated, low-risk pregnancies. The exclusion criteria for participants included strict adherence to medically prescribed special diets for eight weeks before delivery, diarrhea or severe constipation, antibiotic use during the third trimester of pregnancy and postpartum, and consumption of intestinal resorption inhibitors, such as cholestyramine or activated charcoal. Participants were also asked to complete a survey about their lifestyle, diet (focusing on replacing plastic-wrapped foods and beverages, fish, and seafood), and personal care product use from seven days before to seven days after delivery.

### 2.2. MP Detection and Microbiota Analysis from Human Breast Milk Protocol

The protocol for the analysis of MPs and microbiota consisted of six main steps. (a) Breast milk collection: The collection of samples utilized manual expression methods for 10–30 g of breast milk and followed the “Guidelines for Postpartum Care of Mothers and Infants” by the World Health Organization. Clinical Nursing Practice Guidelines [11] were adhered to during the collection process. To minimize the risk of contamination by bacteria and plastics from maternal lifestyle or breastfeeding handling, breast milk was collected within 3 days postpartum using an aseptic protocol at the hospital. Sterile gloves were used, the first few drops were discarded, the breast was thoroughly cleansed with a 2% chlorhexidine solution before manually collecting the breast milk, and the samples were stored at −20 °C [12]. (b) Digestion and filtration of the sample: Using a 10% KOH solution to eliminate organic parts from the milk samples, they were kept at 40 °C for 48 h after being sealed. The digestates were then filtered through a funnel and a vacuum pump with a 1.6 μm pore size membrane. (c) Raman micro-spectroscopy investigation: Morphological characterization of MPs was conducted using an Olympus MPLAN100×/0.90 × 50 objective, followed by Raman micro-spectroscopy analysis on the filter (spectral range 200–1800 cm^−1^, 532, 638, or 785 nm laser diode, 600 lines per mm grating). (d) MP contamination control with an all-plastic-free protocol. (e) Bacterial DNA extraction: Breast milk samples were extracted for bacterial genomic DNA (gDNA). The extraction followed the manufacturer’s protocol for the PrestoTM Mini gDNA Bacteria Kit (Geneaid Biotech, Ltd., New Taipei City, Taiwan). (f) 16S rRNA sequencing and microbiome analysis. The details of each step of the protocol are provided in Supplementary S1.

### 2.3. Sample Size Calculation

The initial sample size calculation, based on the study by Ragusa et al. (2022), indicated that 78 participants were needed to achieve 80% power [9]. This was calculated using the proportions and prevalence formula with a Z-value of 1.96, a significance level of 0.05, and a margin of error of 0.2. However, we recruited 59 participants. Recalculating the power with this sample size resulted in approximately 68.14% power.

### 2.4. Statistical Analysis

Data analysis was performed by using the statistical software package Prism6 (Graph-pad Software, Inc., San Diego, CA, USA). Normality was checked by the D’Agostino and Pearson omnibus normality test. Chi-square test, Student’s *t*-test, and one-way analysis of variance (ANOVA) were performed to compare data accordingly. The significance threshold was set at *p* < 0.05.

## 3. Results

### 3.1. Demographics of the Population

The mean age of the 59 postpartum women was 28.13 years, ranging from 24 to 34 years. A total of 52.54% of participants had a normal BMI, while 38.98% were overweight and obesity, and 8.47% were classified as obese. Most participants (66.10%) had college degrees, while 32.20% had high school degrees. The participants were 38.98% housewives, 20.34% peasants, 13.56% business owners/entrepreneurs, 16.95% salaried workers, and 10.16% students. Pregnancy characteristics included 45.76% primigravida, 55.93% who had vaginal deliveries, and 44.07% who had cesarean sections. The average newborn weight was 3114.91 g. No intrapartum, peripartum, or postpartum complications were reported (Table 1).

### 3.2. Microplastics Detection

**Figure 1:**
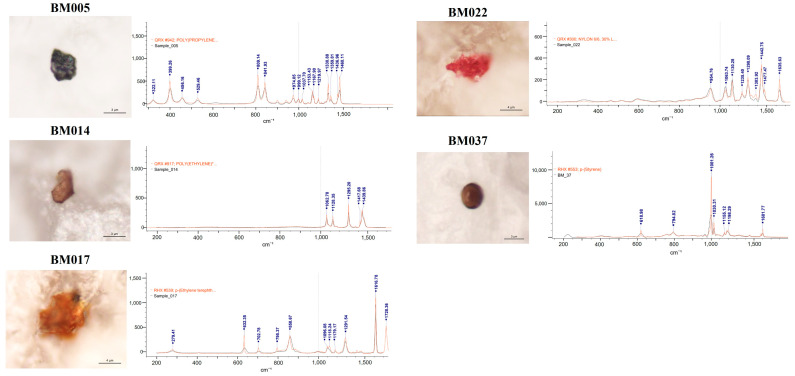
Microphotographs and Raman spectra (wave numbers, cm^−1^) of some selected MPs found in the analyzed breastmilk samples. PE: polyethylene; p-(Ethylene terephthalate); PVC: polyvinyl chloride; PP: polypropylene; NL: Nylon; PS: polystyrene.

MPs were detected in 38.98% (23 of 59) of the breast milk samples. The group with detected MPs included thirteen samples with a single MP polymer, six samples with two MP polymers, three samples containing three MP polymers, and one sample with four MP polymers. The most common MPs were PP, PE, and PVC. The morphological and chemical characteristics of the identified MPs are detailed in Table 2. The analysis revealed a diverse array of MP characteristics within the collected samples. This detailed characterization provided insights into the morphological diversity and chemical composition of the identified microparticles across the collected samples. The detected MPs were identified according to their shape, color, dimensions, and chemical composition (Figure 1). In terms of shape, we identified only irregular fragments and spheres but no films, cellulose, or fibers. Moreover, most of the identified MPs were pigmented (approximately 90%), with dark and brown being the most abundant colors.

### 3.3. Maternal Hygiene Behaviors and Complications during Breastfeeding Compared to the MPs Present and Absent Groups

The hygiene behaviors and breastfeeding complications among mothers were outlined by comparing two groups: those with detected MPs and those without MPs (Table 3). Three categories of maternal hygiene behavior showed statistically significant differences with a higher proportion in the non-detected MPs group: mothers who paid attention to hygiene and washed their hands regularly (88.89% vs. 52.17%, *p*-value = 0.002), mothers who washed their hands after feeding regularly (83.33% vs. 30.43%, *p*-value < 0.001), and mothers who used underwear washing products specifically for mothers and babies (72.22% vs. 17.39%, *p*-value < 0.001).

The breastfeeding complications exhibited noteworthy differences. The MPs-present group experienced a higher proportion with a statistically significant difference in three complications: mastitis (21.74% vs. 0.00%, *p*-value = 0.003), breast engorgement (47.83% vs. 11.11%, *p*-value = 0.002), and low breast milk supply (21.74% vs. 0.00%, *p*-value = 0.003).

### 3.4. *Maternal Behavior on the Risk of Exposure to Chemicals or Products Contaminated with MPs during Current Breastfeeding Compared to MPs Present and Absent Groups*

We elucidated the percentage of maternal behavioral factors associated with MP detection among the breastfeeding participants (Table 4). Eight maternal behaviors showed a statistically significant increase in proportion compared to the non-detected MPs group. These behaviors included skin contact with chemicals (91.30% vs. 62.89%, *p*-value = 0.003), a higher frequency of using plastic packaging for food (91.30% vs. 69.44%, *p*-value = 0.048), a higher tendency to use seafood or shellfish (73.91% vs. 27.78%, *p*-value = 0.001), a higher percentage of mothers who took supplements to stimulate breast milk (47.83% vs. 13.89%, *p*-value = 0.004), and a higher frequency of drinking plastic bottled water (100% vs. 58.33%, *p*-value = 0.001). Other behaviors included frequently drinking tap water (43.48% vs. 8.33%, *p*-value = 0.001), frequently using plastic straws (100% vs. 36.11%, *p*-value < 0.001), and usually cooking food using microwaves in plastic containers (82.61% vs. 13.89%, *p*-value < 0.001). Only one maternal behavior showed a statistically significant increase in proportion in the non-detected MPs group compared to the detected MPs group, which was staying in poor air ventilation during work with chemicals (80.56% vs. 26.09%, *p*-value < 0.001).

### 3.5. Difference of Bacterial Microbiota between MPs Present and Absent Groups

**Figure 2:**
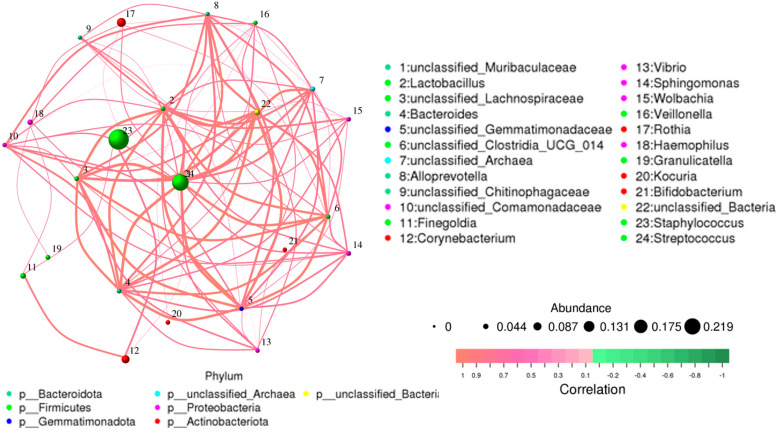
Network map for genus level of all samples. The bacterial abundance of traits was estimated across the entire threshold range (0 to 1, increments of 0.01). The relative abundance of predicted traits was generated for each sample.

**Figure 3:**
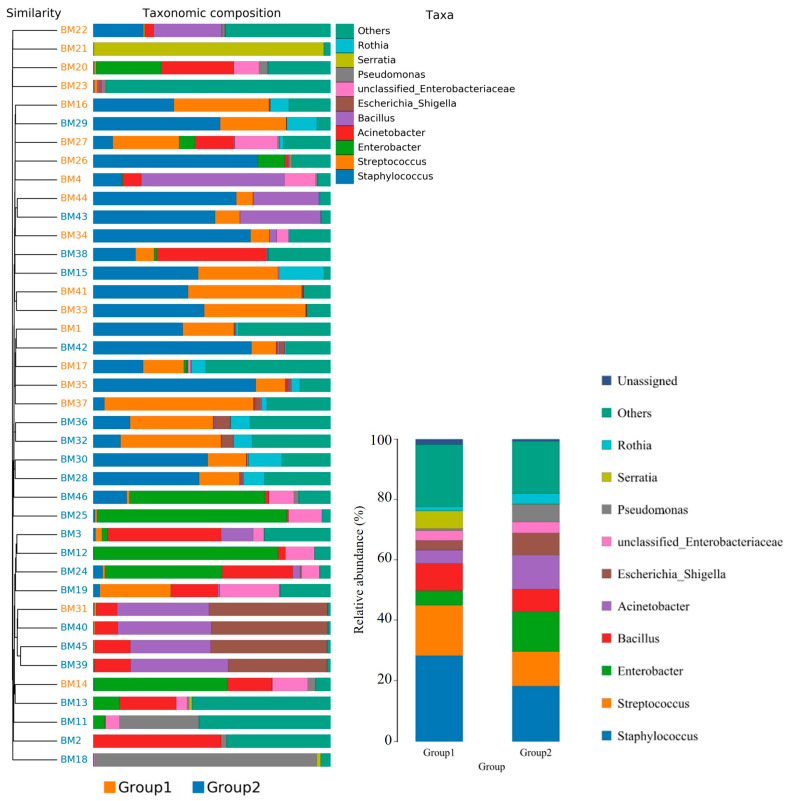
The taxonomic distribution of bacterial communities in breast milk revealed genus-level differences between the group with detected MPs (Group 1) and the group without detected MPs (Group 2). Pairwise comparisons using Wilcoxon test (*p* < 0.05).

Overall, 1286 genera were detected among the breast milk samples in this study. Correlation network analysis illustrated a genus-level network map for all samples, as depicted in Figure 2. Spearman rank correlation analysis was conducted based on the abundance of each species and genus in the samples. The bacterial abundance traits were assessed across the entire threshold range (0 to 1, in increments of 0.01), generating predictions for each sample. Predicted phenotypic types included Gram-positive, Gram-negative, biofilm-forming pathogenic, mobile element-containing, oxygen-utilizing, and oxidative stress-tolerant. The data revealed that most of the relatively abundant bacteria in the breast milk samples comprised five genera: *Staphylococcus*, *Streptococcus*, *Rothia*, *Corynebacterium*, and *Haemophilus*. Gram-positive bacteria, specifically *Staphylococcus* and *Streptococcus*, displayed a significantly higher abundance in Group 1 (presence of MPs), while Group 2 (absence of MPs) exhibited elevated proportions of *Enterobacter*, *Escherichia*, *Pseudomonas*, and A*cinetobacter* (Figure 3).

**Figure 4:**
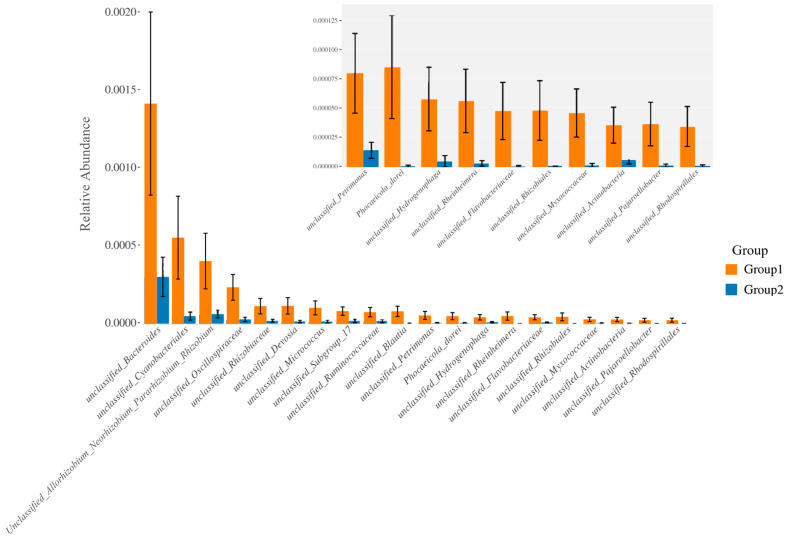
The bacterial species were compared between the group with detected MPs (Group 1) and the group without detected MPs (Group 2). The relative abundance of traits for each sample in the biological dataset was estimated across the entire range of the threshold (0 to 1, in increments of 0.01), shown on the Y-axis. The data were treated by ANOVA analysis.

Furthermore, the relative abundance of traits for each sample in the biological dataset was estimated across the entire range of the threshold (0 to 1, in increments of 0.01). The range from 0 to 1 indicates the full spectrum of possible values for relative abundance, where 0 means the trait is absent and 1 means the trait is fully present. The increments of 0.01 specify the precision of the measurement, with the relative abundance being assessed at each step of 0.01. This approach provides a detailed, granular analysis of how traits are distributed across the samples. The predicted phenotype types included Gram-positive, Gram-negative, biofilm-forming pathogenic, mobile element-containing, oxygen-utilizing, and oxidative stress-tolerant. Figure 4 employed similarity analysis to discern and quantify substantial differences in bacterial abundance at the species level between these two groups. The results underscored the richness of bacterial species by evaluating the relative abundance of individual bacteria within their respective communities. Notably, the evenness of bacterial distribution among species was markedly higher in Group 1 compared to Group 2, particularly within the Bacteroides group. This enhanced evenness suggested different conditions or environments that may have influenced the balanced and diverse microbial community within the breast milk samples of Group 1, underscoring potential ecological implications of the presence of MPs in shaping the bacterial community structure.

**Figure 5:**
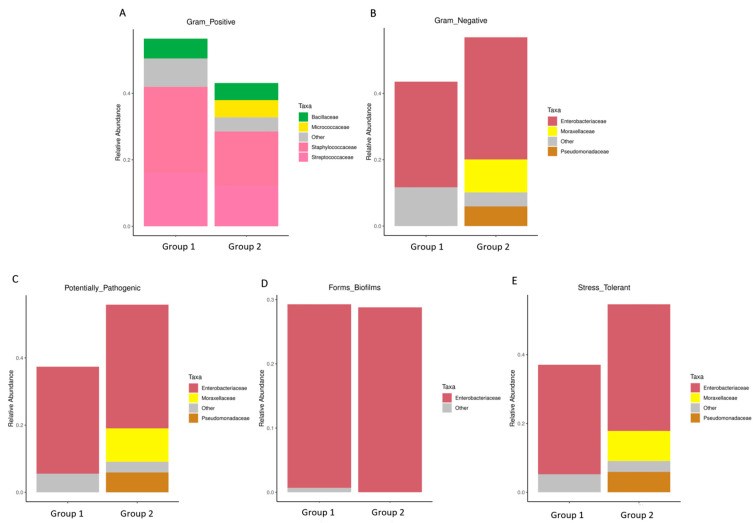
The difference of bacterial phenotype between the group with detected MPs (Group 1) and the group without detected MPs (Group 2). The relative abundance of traits for each sample in the biological dataset was estimated across the entire range of the threshold (0 to 1, in increments of 0.01), shown on the Y-axis. The predicted phenotype types included (**A**) Gram-positive bacteria, (**B**) Gram-negative bacteria, (**C**) potential pathogenic bacteria, (**D**) biofilm-forming bacteria, and (**E**) stress-tolerant bacteria.

Particularly, Figure 5A,B exhibited a higher abundance of Gram-positive bacteria in Group 1, characterized by the prevalence of *Streptococcaceae*, *Staphylococcaceae*, *Micrococcaceae*, and *Bacillaceae*. Conversely, Gram-negative bacteria were more abundant in Group 2, as evidenced by the prevalence of *Enterobacteriaceae*, *Moraxellaceae*, and *Pseudomonadaceae*. Consequently, the proportions of potentially pathogenic bacteria and stress-tolerant bacteria were remarkably dominant in the breast milk samples of Group 2 compared to Group 1. However, no significant difference was observed in the abundance of biofilm-forming pathogens between these two groups (Figure 5C–E).

## 4. Discussion

Overall, the findings of our study indicated that 38.98% of the breast milk samples comprised MPs, with the most prevalent MPs being polyvinyl chloride (PVC), polypropylene (PP), and polyethylene (PE). The process of identifying MPs is carried out using a reliable and credible technique, specifically micro-spectroscopy. This technique permits the direct deposition of particles as small as micrometers onto membranes. This facilitates the examination of morphological characteristics, such as the pigments’ chemical composition and the polymer matrix [13]. The findings of this research are consistent with the preliminary reported detection of MPs in human breast milk in 2022 [9]. However, the sample sizes may have underestimated microparticle detection. Morphological analysis revealed a variety of MP configurations, with irregular fragments and spheres being the most common. MPs could originate from various sources because breast milk lacks films and fibers. PE, PVC, and PP are all petroleum-based plastics, but PE is ideal for food wrap, bottles, and plastic bags. PVC is used for vinyl flooring and plumbing pipe components. PP is ideal for food containers and bottling caps due to its heat resistance [14]. The propensity of these polymers to enter the human body through various routes, including dermal absorption, respiratory inhalation, and ingestion, is well-documented, considering their pervasive presence in our environment [15]. In contrast to our findings, a previous study [10] found MPs in seven of eighteen human breast milk samples, mostly polyurethane (PU) and polyamide (PA). Our study used Raman micro-spectrometry, while that study used an Agilent 8700 Laser Direct Infrared (LDIR) imaging spectrometer, which has limited chemical specificity and may struggle to distinguish similar polymers, especially major plastics like PE, PVC, and PP [16].

The results of this study suggest a possible association between maternal hygiene behaviors and MP detection. Mothers who adhered to good hygiene practices, including frequent handwashing, handwashing after feeding, and using dedicated underwear washing products, were significantly less likely to detect MPs. Despite the lack of any prior research examining the link between MP detection and maternal hygiene practices until now, the possible effects on newborns from MP contamination are not known; healthcare providers should actively educate mothers about the importance of hand hygiene, handwashing after feeding, and using dedicated underwear washing products during the breastfeeding period, which is also beneficial to general health [17]. This research also reports the possible correlation between detected MPs and complications of lactation, namely mastitis, breast engorgement, and decreased milk production. MPs may, for instance, interfere with the normal functioning of the mammary organs, potentially resulting in reduced milk production and an elevated susceptibility to infection [18]. Moreover, MPs may impede the ability of mothers to effectively breastfeed, potentially resulting in additional complications. MP intake significantly impacts the mutualistic relationship between hosts and the bacterial community, leading to a disruption known as dysbiosis. Dysbiosis can induce adverse consequences for the immune system of the host, including but not limited to chronic diseases, heightened susceptibility to pathogenic infections, and alterations in the genetic potential and expression of the bacterial growth or normal flora [19,20].

Personality traits contribute to MP detection in human secretions. Recent studies suggest MPs can enter the body through ingestion, inhalation, skin contact, and other routes [6]. The correlation between maternal behaviors and MPs in breastmilk suggests that these behaviors may indirectly contribute to MP accumulations. According to research, MPs may be absorbed indirectly by the epidermis through personal care products [21]. Mothers who consumed lactation supplements had a higher percentage of MPs in their breast milk than those who did not. The exact mechanisms by which these supplements may contribute to MPs in lactation are unknown, so caution and concern should be considered in advance. Multiple research studies have provided evidence of the existence of MPs in specific food and medical packaging, both of which have the potential to be ingested by humans [22]. Plastic particles from straws, tap water, and bottled water may be a major MP source. MPs have been found in rivers, lakes, groundwater, and tap water, raising concerns about drinking water safety [23]. Furthermore, heat and pressure during microwaving may release MPs from plastic containers, which may accumulate in the mother’s body after ingestion. Plastic degradation during microwave cooking contributes significantly to MP contamination [24]. Microwave radiation breaks down plastic, creating MPs and smaller particles [25]. Small-molecule compounds then form from these particles [26,27].

Our study provides insights into how MPs in breast milk affect the bacterial community. MP-induced alterations in bacterial composition have been reported recently [28]. The significantly higher abundance of Gram-positive bacteria, *Staphylococcus* and *Streptococcu*s, in the group with detected MPs has intriguing implications. The possible mechanisms of the bacteria behind this association could involve the biofilm formation and adhesion capabilities of Gram-positive bacteria to the MP surfaces [25,29]. Whereas the group with an absence of detected MPs displayed a contrasting pattern, with an increased abundance of Gram-negative bacteria, particularly *Enterobacteriaceae*, *Moraxellaceae*, and *Pseudomonadaceae*. This observation suggests a potential influence of MP presence on the selective growth of specific bacterial taxa [25,30]. The results demonstrated that polystyrene (PS) particles could inhibit the cell growth of Gram-negative *Escherichia coli* [25,31]. The study reported a decrease in *E. coli* cell viability with increasing PS concentrations, while no growth inhibition was observed for Gram-positive bacteria such as *Bacillus cereus*. These differences can be attributed to the varying charges of extracellular polysaccharide surfaces (EPS) and the hydrophobicity of bacterial cells, which influence their adsorption on the plastisphere. This study supports our finding that exposure to MPs might contribute to the differing proportions of Gram-positive and Gram-negative bacteria observed in this study. The responses of various bacteria to exposure to MPs can be attributed to the differences in the compositions of their cell walls and the distinct interactions between bacterial groups and PS particles [25,32]. Additionally, the dispersity of PS can be affected by NaCl, other inorganic ions, and natural organic matter. Aggregation of PS may occur if steric and electrostatic repulsion among PS particles is disrupted by either EPS or cells [10]. Gram-negative bacteria have a thinner peptidoglycan layer and an outer membrane composed of lipopolysaccharides, which may facilitate greater interaction with hydrophobic MPs due to their affinity for non-polar surfaces [25,32]. Conversely, Gram-positive bacteria possess a thicker peptidoglycan layer, which may provide a protective barrier against MP-induced stressors [10]. These findings suggest that the varying structural characteristics of bacterial cell walls and the surface properties of MPs play crucial roles in determining bacterial responses to MP exposure. Moreover, elucidating these interactions can help uncover potential pathways through which MPs may exert biological effects. For example, MPs could potentially disrupt microbial communities in body fluids, leading to dysbiosis or altering the metabolic products present in human milk lipids. Understanding these mechanisms is essential for assessing the broader health implications of MP exposure, especially in vulnerable populations such as infants who rely heavily on breast milk for nutrition and immune support. Therefore, future research should focus on characterizing the dynamics of bacterial interaction with MPs in body fluids, including breast milk, to better comprehend their impact on human health and well-being.

While previous literature has documented the presence of MPs in human breast milk, our study further supports the likelihood of detecting MPs in this biological fluid. This discovery holds substantial implications for future research. Moreover, our study rigorously adhered to a participant selection protocol that specifically focused on pregnancies at low risk. Furthermore, our study investigates the potential relationship between detecting MPs in human breast milk and maternal behavior as well as postpartum complications. This study is the first to report an association between MPs and microbiota, initiating further investigations into how microplastics affect microbiota and their mechanisms and effects. However, our study has limitations, including its pilot status with a small sample size, necessitating further evaluation. The recalculated power of 68.14%, due to the reduced sample size of 59 participants, indicates a higher likelihood of a Type II error. This reduction in power suggests that the study may be less likely to detect a significant effect if one exists, which should be considered when interpreting the results. Future studies should aim to meet the initially calculated sample size to ensure sufficient power and more robust findings. Despite this limitation, the current study provides valuable insights into the prevalence of MPs in the population. Moreover, the investigation into detecting MPs using various techniques aims to identify potential sources of MPs in each sample.

## 5. Conclusions

In conclusion, this study reveals that MPs were identified in 38.98% of breast milk samples using Raman micro-spectrometry, with PP, PE, and PVC being the most prevalent types. Significant differences in maternal hygiene practices and breastfeeding complications were observed between the groups with and without detected MPs. The presence of MPs was linked to maternal hygiene, particularly hand hygiene, and behaviors that increase exposure to chemicals or MP-contaminated products, such as food packaging and frequent seafood consumption. Additionally, the detection of MPs may be related to the presence of microbiota in breast milk. This study provides comprehensive insights into the intricate relationships between MP exposure, maternal behaviors, breastfeeding complications, and the composition of bacterial microbiota in breast milk.

## Figures and Tables

**Table 1:** Demographic information of the maternal characteristics.

Participant’s Information	N = 59	%
Age (year), mean (SD)	28.13 (7.93)	
BMI		
- normal - overweight - obesity	31 23 5	52.54 38.98 8.47
Education, n (%)		
High school/college	19	32.20
Degree	39	66.10
Graduated school	1	1.70
Numbers of pregnancy		
Primigravida	27	45.76
Multigravida	32	54.24
Route of delivery		
Vaginal delivery	33	55.93
Cesarean section	26	44.07
Occupation		
Housewife	23	38.98
Peasant	12	20.34
Business/Entrepreneur	8	13.56
Salaries/Waged	10	16.95
Student	6	10.16

**Table 2:** Morphological and chemical characteristics of the identified microparticles in 23 samples.

Sample Code No.	Age	Milk Quantity (g)	Number of MPs	Shape	Color	Polymers Identified
BM001	23	15.0	2	irregular fragment	dark brown	polyvinyl chloride
				sphere	brown	polyvinyl chloride
BM005	45	14.3	1	irregular fragment	black	polypropylene
BM014	17	10.5	1	irregular fragment	brown	polyvinyl chloride
BM016	28	15.0	1	irregular fragment	black	polystyrene
BM017	18	10.0	3	irregular fragment	green	p-(Ethylene terephthalate)
				irregular fragment	yellow	polyethylene
				irregular fragment	dark brown	polyvinyl chloride
BM020	22	10.0	1	irregular fragment	blue	polyethylene
BM021	45	15.0	1	irregular fragment	brown	polyethylene
BM022	48	15.0	2	sphere	brown	polyvinyl chloride
				irregular fragment	pink	nylon
BM023	38	12.0	1	irregular fragment	red	polyvinyl chloride
BM026	31	12.0	1	sphere	dark brown	polystyrene
BM027	19	12.0	4	irregular fragment	brown	polypropylene
				irregular fragment	green	polyethylene
				sphere	brown	polypropylene
				irregular fragment	brown	polypropylene
BM031	28	12.0	2	irregular fragment	brown	polypropylene
				sphere	brown	polypropylene
BM033	23	12.0	1	irregular fragment	brown	polyethylene
BM034	43	10.3	1	irregular fragment	brown	polypropylene
BM035	22	11.5	1	irregular fragment	green	polyethylene
BM037	41	11.0	2	sphere	dark brown	polystyrene
				irregular fragment	blue	polyvinyl chloride
BM041	36	10.0	2	irregular fragment	green	polyethylene
				irregular fragment	gray	polypropylene
BM043	36	10.0	3	irregular fragment	brown	polypropylene
				sphere	dark brown	polystyrene
				irregular fragment	brown	polyethylene
BM044	32	10.0	1	irregular fragment	brown	polypropylene
BM046	19	15.0	1	irregular fragment	red	polyvinyl chloride
BM049	21	15.0	3	irregular fragment	green	polyethylene
				irregular fragment	brown	polypropylene
				irregular fragment	gray	polypropylene
BM051	23	12.0	2	irregular fragment	brown	polypropylene
				irregular fragment	brown	polyethylene
BM053	34	12.0	1	irregular fragment	brown	polypropylene

**Table 3:** Percentage of maternal hygiene behaviors and complications during breastfeeding compared between groups with and without MPs.

Maternal Characteristics	Group 1 (n = 23) MPs Present (Percentage)	Group 2 (n = 36) MPs Absent (Percentage)	*p*-Value
**Hygiene behaviors**			
Usually drink sterilized milk or beverages	47.83	52.78	0.711
Pay attention to hygiene and wash your hands regularly	52.17	88.89	0.002 *
Wash hands before feeding regularly	78.26	91.67	0.142
Wash hands after feeding regularly	30.43	83.33	<0.001 *
Clean breasts with soap regularly	56.52	69.44	0.312
Always dry your breast thoroughly after washing	21.74	41.67	0.141
Use underwear washing products specifically for mothers and baby	17.39	72.22	<0.001 *
**Complications**			
Clogged milk ducts	21.74	8.33	0.926
Mastitis	21.74	0.00	0.003 *
Abscess	8.70	0.00	0.072
Breast engorgement	47.83	11.11	0.002 *
Low breast milk supply	21.74	0.00	0.003 *

* Statically significant (*p*-value < 0.05).

**Table 4:** Percentage of maternal behavior regarding the risk of exposure to chemicals or products contaminated with microplastics during current breastfeeding compared between groups with and without MPs.

Maternal Behavior	Group 1 (n = 23) MPs Present (Percentage)	Group 2 (n = 36) MPs Absent (Percentage)	*p*-Value
Smoking during pregnancy or breastfeeding	0.00	0.00	-
Work in a profession or place related to regular exposure to chemicals	52.17	33.33	0.151
Regular contact with chemicals in the residence	69.57	44.44	0.059
Stay in bad air ventilation during work with chemicals	26.09	80.56	<0.001 *
How to contact the chemicals			
Inhalation	65.22	30.56	0.009
Licking or swallowing	21.74	5.56	0.061
Skin contact	91.30	63.89	0.003 *
Exposure to car soot, combustion, and air pollution	82.61	41.67	0.012
Use cosmetics or hair spray	60.87	30.56	0.022
Get nails manicured at a salon or use manicure products	26.09	11.11	0.135
Use a breast pump	39.13	33.33	0.650
Frequently use plastic packaging for food	91.30	69.44	0.048 *
Usually have seafood or shellfish	73.91	27.78	0.001 *

* Statically significant (*p*-value < 0.05).

## Data Availability

Data are contained within the article and Supplementary Materials.

## Supplementary Materials

The following supporting information can be downloaded at: https://www.mdpi.com/article/10.3390/jcm13144029/s1. Supplementary S1: data supplemental to the main text—explanations of experimental details.
