# Supplementary material for: Detection of Microplastics in Human Breast Milk and Its Association with Changes in Human Milk Bacterial Microbiota
Source: J Clin Med. 2024 Jul 10;13(14):4029. doi: 10.3390/jcm13144029 (PMC11277308; doi:10.3390/jcm13144029)
Supplement: Supplementary file 1 [file jcm-13-04029-s001.zip › jcm-3038135-supplementary.pdf]

Supplementary S1: data supplemental to the main text—explanations of experimental details.

MPs detection and microbiota analysis from human breast milk protocol

a. Breast milk collection

The protocol required participants to provide breast milk samples within one week after delivery. To minimize the risk of plastic contamination from maternal lifestyle or breastfeeding handling, 10-30 grams of breast milk were collected within 3 days postpartum at the hospital. The protocol strictly controlled for bacterial contamination and plastics, following the recommendations of the Lactation Nurses Association of Thailand (LNAT) and the 'Guidelines for Postpartum Care of Mothers and Infants' by the World Health Organization. Prior to collection, participants were informed about the procedure, provided consent, and were instructed on proper practices by a professional nurse (a single-nurse protocol) at the same breast milk clinic. Samples were collected using manual expression methods under an aseptic protocol (Jung et al., 2022). Sterile gloves were worn, the first few drops of milk were discarded, and the udder was thoroughly cleansed with a 2% chlorhexidine solution before manual collection. Breast pumps were avoided to reduce plastic contamination. Manual self-expression involved cupping the breast with one hand and making a C shape with the thumb and index finger 3-4 cm from the nipple base. The breast milk samples were placed in glass vials and stored at -20 °C until use. Each sample was divided into two portions for MPs analysis and microbial genetic extraction."

b. Digestion and filtration of the sample

In order to eradicate organic constituents from samples of milk, the Food Analytical Laboratory developed and implemented a digestion protocol. In each flask, 1.6 m filtered deionized water was combined with 1:10 (w/v) KOH tablets (Sigma-Aldrich) to produce a 10% KOH solution. The flasks were incubated at 40 °C for a duration of 48 hours subsequent to their sealing. Following incubation, the digestates underwent filtration via a funnel and vacuum pump through a Whatman GF/A pore-size filter membrane with a pore size of 1.6 µm. The filter membranes were placed in glass Petri dishes and allowed to dry naturally at room temperature prior to undergoing Fourier Transform Raman Spectroscopy (FT-Raman) analysis.

c. Raman microspectroscopy detection

RMS analysis was conducted utilizing an FT-Raman Microspectrometer (PerkinElmer® Spectrum™ GX systems). A visible light examination was performed on all filter membranes, including procedural blanks, using an Olympus MPLAN10/0.25 instrument. Morphological characterization of MPs was conducted using an Olympus MPLAN100×/0.90 ×50 objective, followed by Raman Microspectroscopy analysis on the filter (spectral range 200-1800 cm−1, 532, 638, or 785 nm laser diode, 600 lines per mm grating). The spectrum quality and noise reduction of the raw Raman spectra were achieved through the utilization of Labspec 6 (Horiba Scientific), which performed vector normalization and polynomial baseline correction. By comparing Raman spectra to polymer and pigment spectral libraries obtained from standard polymers/compounds (KnowItAll software, John Wiley & Sons, Inc., Hoboken, NJ, USA), the polymer matrix of the identified particles could be identified. A Hit Quality Index (HQI) similarity exceeding 80 was deemed satisfactory.

d. MP contamination control

A systematic plastic-free protocol decreased the risk of MPs contamination. Sterilized glass was utilized in place of plastic tools. The experimenters consistently utilized cotton lab coats and single-use latex gloves. Filtration was performed on all liquids, ethanol being used for cleaning and deionized

water being employed for solution preparation, through Whatman GF/A filter membranes with a pore size of 1.6 μm. Prior to commencing procedures and for the duration of the experiment, all work surfaces were thoroughly cleaned using 70% ethanol. Stainless steel tweezers and scissors were among the implements that were sanitized with dishwashing liquid, followed by three rinses with 70% ethanol and a final rinse with 1.6 m filtered deionized water. Sources of MPs contamination were also thoroughly investigated. In order to include environmental blanks in the research, an uncovered Petri dish was placed daily in the designated room containing a filter membrane saturated in 1.6 μm filtered deionized water. In the same rigorous manner as the preparation of each sample batch, procedural blanks were meticulously prepared in the absence of milk. Environmental and procedural blank filters were examined with a stereomicroscope to ensure the absence of MPs.

e. Bacterial DNA extraction

Breast milk samples were extracted for bacterial genomic DNA (gDNA). The extraction followed the manufacturer's protocol for the Presto™ Mini gDNA Bacteria Kit (Geneaid Biotech, Ltd., New Taipei City, Taiwan). Resuspend the pellet with 200 μL of lysozyme and incubate at 37°C for 30 minutes. After removing the supernatant, 20 μL of proteinase K was added and incubated at 60°C for 10 minutes. DNA bound to GD column after lysis. Washing and eluting gDNA in the collection tube. One microcentrifuge tube was used to collect purified gDNA, which was centrifuged at 15,000×g for 30 seconds. The concentration of gDNA was measured using Spectrophotometric Nanodrop and stored at -20 ºC until library construction.

f. 16S rRNA sequencing, microbiome analysis

The Center of Biomedical Analysis, developed a method to amplify the V3 region of the 16S rRNA gene using 10 ng of precipitate and molecular barcodes for multiplex sequencing per sample. The amplicons were sequenced using a desktop sequencer (MiSeq; Illumina, Inc., San Diego, CA, USA) using paired-end sequencing (2x150 bp). To maximise data resolution, the 16S rRNA gene pipeline uses phylogenetic and alignment-based methods. Read pairs were demultiplexed using unique molecular barcodes and merged using USEARCH v7.0.1090 [20] with at least a 50-bp overlap and no more than a 1-bp mismatch. The UPARSE algorithm clustered merged sequences into OTUs at 97% similarity. A 0.5 expected error rate was used for quality filtering. Taxonomies were determined by mapping OTUs to SILVA (version 123). DADA2 (v1.16.0 pipeline) calculated alpha diversity index (Chao1 richness, Shannon, and PD whole tree). From Phyloseq data, Bray-Curtis dissimilarity and PCoA were plotted for Bata diversity. To find bacterial biomarkers, LEFSe and cladogram plots were used. To avoid bias during sample collection and processing, we excluded OTUs from QIIME blanks.
